# Schnell wachsende Raumforderung der Gl. parotidea ohne Fazialisparese

**DOI:** 10.1007/s00106-024-01459-2

**Published:** 2024-04-09

**Authors:** Sheila Büchel, Markus Jungehülsing

**Affiliations:** Klinik für Hals‑, Nasen- und Ohrenheilkunde, Ernst-von-Bergmann-Klinikum, Charlottenstraße 72, 14467 Potsdam, Deutschland

## Anamnese

Wir berichten über eine 47-jährige Patientin, die sich erstmals im Juni 2023 mit einer schnell wachsenden Raumforderung der rechten Gl. parotis vorstellte. Die Raumforderung bestand seit 6 Monaten, die Fazialisfunktion war regelrecht. In der Sonographie waren eine 4 cm messende, glatt berandete, echoarme, homogene Struktur in der zentralen Parotis rechts sowie zwei rundliche Lymphknoten ohne Binnenstruktur und mit fehlendem Hilus in Regio III schallbar.

Bei Verdacht auf eine Lymphomerkrankung erfolgte die Stanzbiopsie, in der sich der Verdacht nicht bestätigte.

### Erhobene Befunde.

**Abb. 1:**
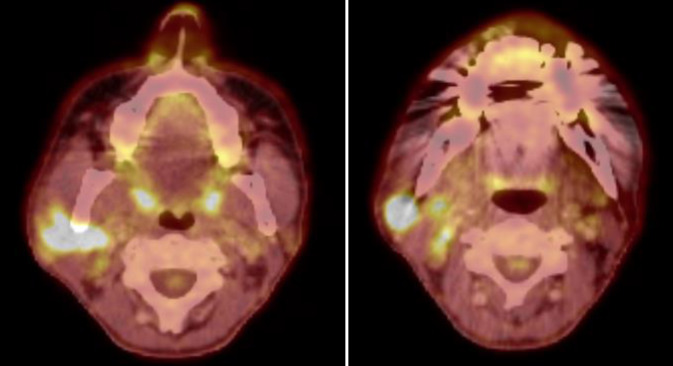
18-FDG-PET-Fusions-CT vom Juni 2023

18-Fluordesoxyglucose-Positronenemissionstomographie-Fusions-Computertomographie (18-FDG-PET-Fusions-CT) und Ganzkörper-18-FDG-PET vom Juni 2023 zeigen den Primärtumor und die Hals-Lymphknotenmetastasen, jedoch keine Fernmetastasierung (Abb. 1 und 2).

**Abb. 2:**
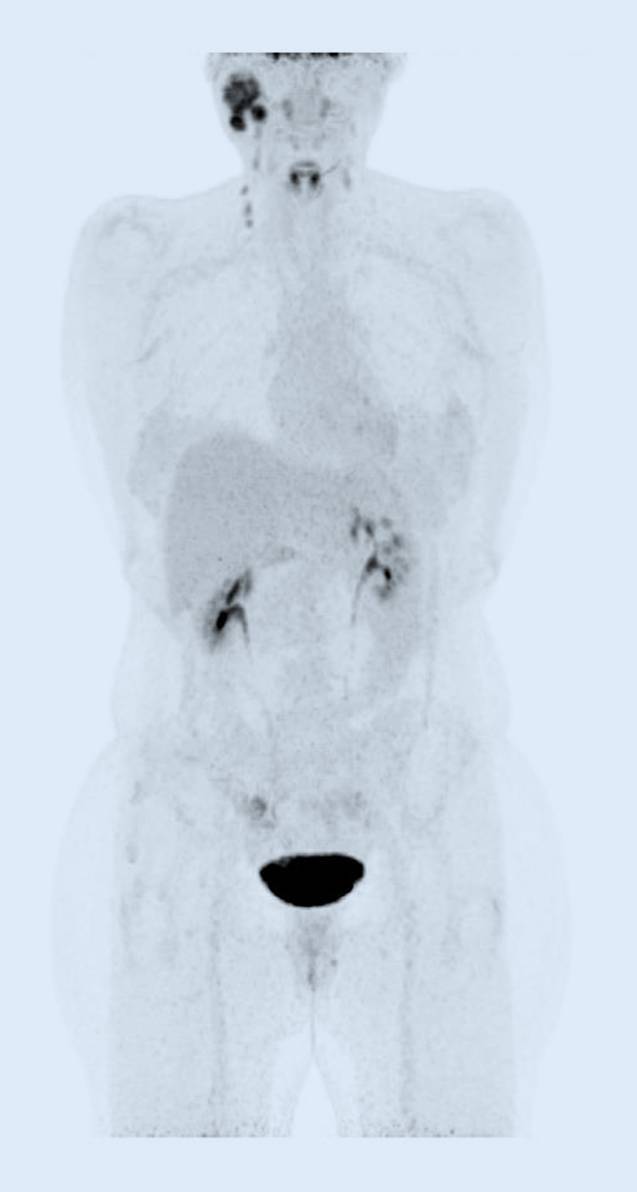
Ganzkörper-18-FDG-PET mit Primärtumor und Halslymphknotenmetastasen, ohne Nachweis von Fernmetastasen vom Juni 2023

### Laborparameter vom Juni 2023.

Normalbefund

## Wie lautet Ihre Diagnose?

**Diagnose: **NUT-Karzinom der Gl. parotis rechts mit NUTM1-Fusion

## Therapie und Verlauf

**Abb. 3:**
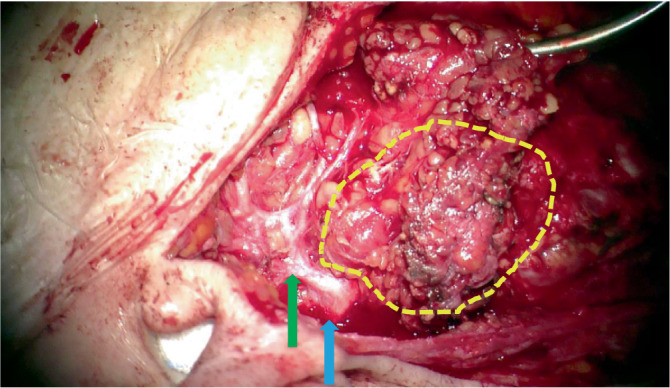
Intraparotideal gelegener Tumor: *gelb* umrandet: palpabler, teilweise sichtbarer Tumor; *grüner Pfeil*: frontofazialer Hauptast N. facialis; *blauer Pfeil*: Fazialisstamm

Bei Nachweis eines NUT-Karzinoms in der Stanzbiopsie erfolgte eine totale Parotidektomie rechts mit Resektion des infiltrierten zervikofazialen Hauptasts des N. facialis und Rekonstruktion mit einer HFJ-Anastomose sowie eine modifizierte radikale Neck-Dissection rechts (Abb. 3 und 4).

**Abb. 4:**
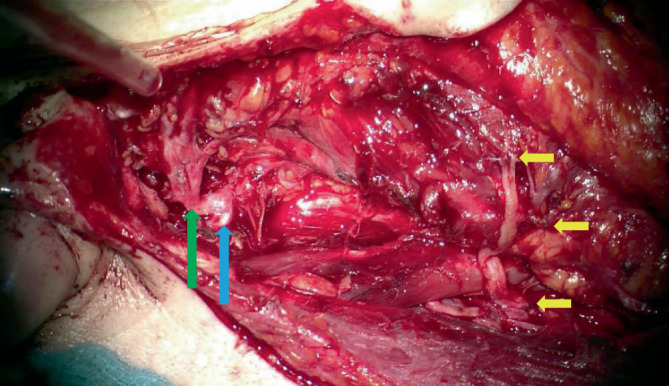
Diversifikation nach Stennert: Fazialisstamm (*blauer Pfeil*) und frontofazialer Hauptast (*grüner Pfeil*) erhalten, zervikofaziale Fazialisäste über N. auricularis magnus mit dem N. hypoglossus Seit-zu-End-anastomosiert (*gelbe Pfeile*)

Intraoperativ zeigte sich mikroskopisch wie histologisch in multiplen Schnellschnitten eine Infiltration des zervikofazialen Hauptasts des N. facialis durch den darunterliegenden Tumor, sodass der Fazialisast mit der Glandula parotidea und dem Tumor reseziert und gemäß Schnellschnittergebnissen weit peripher nachreseziert werden musste, um eine R0-Situation zu erreichen. Infiltrationen anderer umgebender Strukturen fanden sich weder mikroskopisch noch in den Schnellschnitten. Nach Komplettierung der Neck-Dissection (Regio II bis V nach Robbins und Medina) wurde ein N.-auricularis-magnus-Transplantat gewonnen. Wegen der Länge des entstandenen Fazialisdefekts gelang keine direkte Anastomose zwischen der Bifurkation und den peripheren Nervenenden des Fazialis mit dem N. auricularis magnus. Deswegen wurde der zervikofaziale Hauptast im Sinne einer Hypoglossus-Fazialis-Jump-Anastomose (HFJA) über den N. auricularis magnus mit dem N. hypoglossus anastomosiert.

## Histologie

In der initialen Histologie fand sich eine schlecht differenzierte maligne epitheliale Neoplasie mit abrupter plattenepithelialer Differenzierung, suspekt auf das Vorliegen eines NUT-Karzinoms. In einer Referenzpathologie zeigte sich sowohl in der Stanze als auch im Tumorpräparat im TruSight-RNA-Fusionspanel eine NSD3: NUTM1-Fusion [4] und damit ein gewöhnliches NUT-Karzinom.

Insgesamt ergab sich so folgende Tumorformel: pT4a, pN2b (7/32, ECE-), cM0, L0, V1, Pn0, R0.

Bei undifferenzierten Parotismalignomen führen wir regelhaft ein immunhistochemisches Screening auf Hormonrezeptoren, epidermale Wachstumsfaktorrezeptoren und Immunmodulatorenrezeptoren durch. Dies ist der Tatsache geschuldet, dass undifferenzierte Malignome (neuroendokrine Karzinome, High-Grade-Mukoepidermoidkarzinome, Azinuszellkarzinome, myoepitheliale Karzinome) [5] diese gelegentlich exprimieren und damit näher eingegrenzt und auch erfolgreicher therapiert werden können. Die Ergebnisse für Östrogen- und Progesteronrezeptoren, Androgenrezeptoren, HER2 und PD-L‑1 waren allerdings negativ.

## Verlauf

Der postoperative Befund zeigte eine Parese der Unterlippe und des Platysmas, die aktuell (8 Monate nach Operation) regredient sind.

Bei aggressivem Tumorverhalten und nur knapp reseziertem Tumor wurde eine Radiochemotherapie (ad 64 Gy auf die Parotisregion und ad 54 Gy auf die Lymphabflusswege, 1 x 40 und 3 x 50 mg/m^3^ Cisplatin wöchentlich) im Tumorboard empfohlen. Diese wurde von Juni bis August 2023 durchgeführt und bis auf eine leichte Strahlenmukositis mit Thrombozytopenie gut toleriert. Zusätzlich erfolgte die Anbindung an das Comprehensive Cancer Center der Charité Berlin.

Die Patientin wurde im weiteren Verlauf in die Tumorsprechstunde angebunden. Aktuell (6 Monate nach Ende der Therapie) zeigt sich eine kleine nässende Fistel an der Narbe subaurikulär sowie ein Frey-Syndrom, welches im Verlauf mit Botox (20 U Xeomin® intrakutan im sezernierenden Bereich) behandelt wird. In der im Januar 2024 durchgeführten PET-CT-Kontrolle konnten 6 Monate nach erfolgter Therapie keine neuen stoffwechselaktiven Herde nachgewiesen werden, sodass die Patientin sich aktuell in Remission befindet. Eine erneute Staging-CT ist 9 Monate nach Therapieende geplant.

## Definition

Erstmals wurde das NUT-Karzinom 1991 in Boston beschrieben. Es handelt sich um ein schlecht differenziertes Plattenepithelkarzinom, dass durch ein chromosomales Rearrangement auf dem Chromosom 15 erklärbar ist. In 2/3 der Fälle wird eine Fusion zwischen NUT und BRD4 (Chromosom 19) beschrieben, in den weiteren Fällen handelt es sich um eine Fusion des NUT-Gens mit BRD3 (Chromosom 9) oder mit anderen Genen. Diese blockiert die Zelldifferenzierung und hält die Tumorzelle in einem hochproliferativen, undifferenzierten Stadium. Möglicherweise wird auch die MYC-Expression dereguliert, welche dann zu einer verstärkten Expression von wachstumsrelevanten Genen führt [1].

NUT-Karzinome, früher NUT-Mittellinienkarzinome, sind sehr aggressiv und können alle Altersgruppen befallen. Häufig werden sie im Aerodigestivtrakt gefunden, können aber auch abdominal auftreten [2].

Dabei sind bisher nur wenige parotideale NUT-Karzinome beschrieben [4, 5]. Das mediane Überleben beträgt zwischen 6 und 9 Monaten. Die Tumoren sprechen schlecht auf eine Radiochemotherapie an [3].

Primäre Plattenepithelkarzinome (PEC) der Parotis sind sehr selten (0,1–10 % der Parotismalignome). Wie häufig es sich dabei um ein NUT-Karzinom handelt, ist unbekannt. Fakt ist, dass man bei primären Plattenepithelkarzinom (PEC) der Parotis auch an einen NUT-Tumor denken sollte. Vorher sollte der häufigste maligne Tumor der Parotis, die Metastase eines Haut-PEC, ausgeschlossen werden [4, 5].

## Fazit für die Praxis

- Beim sehr seltenen primären Plattenepithelkarzinom der Parotis kann es sich um ein NUT-Karzinom handeln.
- Eine NUTM1-Fusion sollte ausgeschlossen werden.
